# Acceptability of testing for anorectal sexually transmitted infections and self-collected anal swabs in female sex workers, men who have sex with men and transgender women in Papua New Guinea

**DOI:** 10.1186/s12889-018-5684-2

**Published:** 2018-06-20

**Authors:** Stephen Bell, Johanna Wapling, Sophie Ase, Ruthy Boli-Neo, Andrew J. Vallely, John M. Kaldor, Claire E. Nightingale, Angela Kelly-Hanku

**Affiliations:** 1 The Kirby Institute, UNSW Sydney, Sydney, Australia; 2 Centre for Social Research in Health, UNSW Sydney, Sydney, Australia; 3 Sexual and Reproductive Health Unit, Papua New Guinea Institute of Medical Research, Goroka, Eastern Highlands Papua New Guinea

**Keywords:** Anorectal STIs, Acceptability, Anorectal STI testing, Key populations, Self-collection, Papua New Guinea

## Abstract

**Background:**

Papua New Guinea (PNG) has some of the highest prevalence of urogenital sexually transmitted infections (STIs) in Pacific Asia, but to date, anorectal STI prevalence data do not exist, and diagnosis of anorectal STIs does not occur. The purpose of this study was to document the acceptability of anorectal STI testing and self-collection of anorectal swabs for testing among populations at risk of anorectal STIs, in advance of a large bio-behavioural survey during which this approach to specimen collection was planned among key populations in PNG.

**Methods:**

Four focus groups were conducted, collecting data from a purposive sample of 35 members of two civil society groups representing female sex workers, men who have sex with men and transgender women in Port Moresby and Goroka.

**Results:**

All participants were in favour of anorectal STI testing in PNG. Reasons given for willingness to undertake anorectal STI testing included that anal sex is practised; that anorectal STIs are not perceived to exist; there are self-reported experiences of anorectal symptoms indicative of anorectal STIs; that anorectal STI testing will enhance personal health; and that anorectal STI testing is not currently available in PNG. All participants were confident they could obtain self-collected specimens, although several stated that support from trained health workers should be available for community members who may not feel comfortable with self-collection.

**Conclusions:**

This qualitative research is the first study of acceptability of anorectal STI testing and specimen self-collection procedures in PNG, and Pacific Asia more broadly. Our qualitative findings show support for anorectal STI testing including the use of self-collected swabs among key populations in PNG. Study findings informed the inclusion of anorectal STI testing in a large bio-behavioural survey to be used to estimate anorectal STI prevalence among key populations in PNG for the first time.

## Background

Sexually transmitted infections (STIs) are a serious global public health issue, especially for vulnerable and disadvantaged communities. According to latest World Health Organisation data, in 2012 alone, there were an estimated 357 million new global cases of curable STIs (including chlamydia, gonorrhoea, trichomoniasis and syphilis, for example), 142 million of which were in the Western Pacific Region [1]. Papua New Guinea (PNG) is described as having some of the highest prevalence of urogenital STIs in the region [2–5]. With no access to diagnostic STI testing in PNG – in accordance with WHO syndromic management guidelines, STIs are managed based on clinical presentation without laboratory confirmation [6] – anorectal STI prevalence information does not currently exist.

Despite the lack of anorectal STI prevalence data in PNG, a systematic review of behavioural research in PNG highlighted that heterosexual anal intercourse practices are widespread, and pointed to the likely impact such practices have on STI incidence at the population level [7]. The sexual health risks associated with unprotected penile-anal intercourse are well documented, facilitating the acquisition and transmission of STIs. Bacterial STIs, including gonorrhoea and chlamydia, can colonise the anorectal canal, and are often asymptomatic, so may remain undetected and untreated, representing a risk to sexual partners [8]. Unprotected heterosexual anal intercourse is significantly associated with an increased incidence of vaginal STIs in women [8, 9]. Unprotected anal intercourse is also associated with higher rates of human immunodeficiency virus (HIV) transmission than unprotected vaginal intercourse [10–14].

This qualitative paper reports on findings from the first study undertaken with the purpose of examining the acceptability of anorectal STI testing and specimen self-collection procedures among female sex workers, men who have sex with men and transgender women in PNG.

## Methods

A qualitative research approach was adopted to engage members of ‘key populations’ – groups of people who are key to the dynamics of, or response to, an HIV and STI epidemic [15, 16] – in a pilot investigation of acceptability of anorectal STI testing and specimen self-collection procedures. This pilot study was required prior to integrating anorectal STI testing in a large bio-behavioural survey to ascertain whether members of these populations would entertain the idea of anorectal STI in a national context in which there is no laboratory STI testing, and where anorectal sex is illegal. Given the diverse settings across PNG, this qualitative study adopted an understanding of acceptability that emphasises people’s experiences, meanings and comprehension within specific and dynamic social and cultural contexts [17].

Research was conducted in two locations – Port Moresby, National Capital District, and Goroka, Eastern Highlands Province. These sites were selected as they represent two distinct regions of PNG, and there are existing relationships between the PNG Institute of Medical Research (IMR) and key population networks.

People who self-identified as members of one of three key populations – female sex workers, men who have sex with men, and transgender women – were invited to participate in focus groups. These key population groups are particularly at risk of STIs associated with anal intercourse. In May and August 2015, four focus groups were conducted with female sex workers (3), and men who have sex with men and transgender women (1) (see Table 1). Each focus group was sex specific. In PNG, transgender women advocate change with men who have sex with men, and there is one shared peer-led civil society representing both populations. Therefore, with their approval, transgender women and men who have sex with men were involved in one focus group discussion. A focus group discussion with men who have sex with men and transgender women was sought in Goroka. However, due to stigma in the Highlands Region, we were not able to recruit enough participants for a focus group.

**Table 1:** Research participant information

Focus group	Research participant group	No. of participants	Location
FGD 1	Female sex workers	9	Goroka
FGD 2	Female sex workers	6	Goroka
FDG 3	Men who have sex with men and transgender women	11	Port Moresby
FGD 4	Female sex workers	9	Port Moresby

A purposive sampling technique [18] was used to recruit participants. Inclusion criteria for participants were that they were 18 years or older, a member of a key population, willing to discuss anal sex and anorectal STIs, and could provide informed consent. Recruitment was initiated through snowballing via prominent, well-respected members of these populations who were working with two civil society organisations representing key populations – *Friends Frangipani* (female sex workers) and *Kapul Champions* (men who have sex with men and transgender women) – and who were known to the research team, and who invited other individuals from their social and professional networks to participate in the research.

Focus groups consisted of 5–9 people, lasted between 60 and 90 min, and were conducted in Tok Pisin by IMR social researchers and the last author, and were conducted in settings offering audio-privacy to research participants. Focus group topics explored included: experiences of anal sex and anorectal STIs, knowledge and awareness of anorectal STIs, and new anorectal STI testing processes involving self-collection of anorectal swabs. During these focus group discussions, researchers informed participants about anorectal STIs, symptoms and treatment, and engaged populations in the design and development of culturally appropriate illustrated instructions that could be used to facilitate self-collection of anorectal specimens for STI testing in PNG.

All focus groups were digitally audio recorded, transcribed and translated from *Tok Pisin* to English. All personal identifiers were removed from the focus group transcripts and pseudonyms given to each participant. Data were subjected to rigorous thematic analysis by two researchers – SB and AKH – following a system of inductive ‘open’ and ‘axial’ coding [19] using Nvivo 11 (QSR International). Open coding involves reading through the data to increase familiarity with the material and to record ‘theoretical memos’ [19] as analytical reminders for generating ideas and making links between different data. Axial coding describes the later process of linking or organising open codes into themes and sub-themes, and providing evidence to support thematic findings.

Ethical approval for this study was obtained from the PNG IMR Institutional Review Board (IRB1319), and the PNG National Department of Health Medical Research Advisory Committee (MRAC13.32). Key ethical principles adhered to throughout the investigation include voluntary participation, informed consent, confidentiality and anonymity. All participants provided written informed consent prior to participation.

## Results

### Finding 1: People are having anal sex

Anal sex was perceived as a common practice amongst female sex workers, men who have sex with men and transgender women. Focus group participants also thought that anal sex was common amongst the general population. For these reasons, participants believed that anorectal STI testing should be available for anyone at risk of anorectal STIs.

I used to put it in the mouth or put it in the anus… Anal sex is a normal thing… The straight boys are now having anal sex. The straight men and the women, they do such.

(*Man who has sex with other men, Port Moresby*)

Many people nowadays are having anal sex. Even married men and married women. Our husbands watch those porn [films] so they want to have sex with us from the anus. I think that it is good that you people can do research [and STI testing] on that on us. Check our anus.

*(Female sex worker, Goroka)*

### Finding 2: People have experienced anorectal symptoms

Participants either reported personal experience of anorectal symptoms, or were able to talk about other people they knew who were having anal sex and had experienced such symptoms. Symptoms were indicative of anorectal STIs, were described vividly, and were associated with discomfort.

STIs that usually develop in our anus. Some of us, we usually see that our asshole will become itchy, water will drop and pus will smell. Then we know we have an STI.

*(Transgender woman, Port Moresby)*

It used to be painful and wet, and stinks, and there were like little things like boils. And there was water coming out of the edges and it was really giving off bad smell.

(*Female sex worker, Goroka*)

### Finding 3: There are misconceptions that anorectal STIs do not exist

Despite some shared personal experience of symptoms associated with anorectal STIs, there still existed misconceptions about anorectal STIs among female sex workers. From our data, it appears common that participants, their clients and sexual partners believe that STIs are limited to the genital area. This had consequences for their sexual practices (i.e. condomless anal sex) and sexual health check-ups (genital focus only).

I understood there are no anal STIs. Thank you for asking because before I thought there were no anal STIs. Because I haven’t felt any signs or symptoms so I haven’t felt or seen this. I didn’t know there were anal STIs... Now that you people are talking about it and it has opened my mind.

(*Female sex worker, Goroka*)

Some men believe that diseases are in the vagina only.

(*Female sex worker, Port Moresby*)

### Finding 4: Anorectal STI testing will enhance personal health

Participants acknowledged that they are at risk of anorectal STIs due to their participation in unprotected anal sex. They also explained that the opportunity to be screened and treated for anorectal STIs would enhance their own personal health safety, and enable them to manage the consequences of unprotected anal intercourse.

If I have anal sex then I will still take that one [anal STI test] because I would want to know if I have an STI or something like that at my anus because he had anal sex with me without a condom… Those STIs that we have, the doctor lady tells us to lie on the bed and remove all our clothes. Likewise for the anus we would do it. It’s for our own safety.

(*Female sex worker, Port Moresby*)

I go around secretly and have anal sex and I don’t know if I am sick or not. I am thankful that we had this discussion, it opened my thoughts. It has opened our thoughts to look at the next level of health care that we can look after ourselves. We can look after the vagina, penis, anus or our mouths. This is for our own good.

(*Female sex worker, Port Moresby*)

### Finding 5: Anorectal STI testing is not available in PNG

Participants supported the need for anorectal STI testing because, at present, health care workers only offer to provide syndromic management for vaginal and penile STIs.

Anal sex is there, and there are no places to serve people who have anal sex. We have no clinics for this section, to check the anus. …[The doctors] think that we only have vaginal sex.

(*Female sex worker FGD, Goroka*)

No, [the health workers] did not check our anus but they gave us medicine.

(*Transgender woman, Port Moresby*)

For us that come to the clinic, they used to test us on the hand. It’s not to check us (at the anus). They usually check our hands and they use it to treat us.

(*Man who has sex with other men, Port Moresby*)

It will help us because for STIs we only get medication and we open our legs for them to check [the vagina]. There are no checks that happen for the back [anus]. So this will be alright.

(*Female sex worker, Port Moresby*)

Research participants also indicated that treatment for anorectal STIs is only provided if they are symptomatic. Testing procedures are blood-based, and these are only available at health clinics.

[A friend] said ‘I have a tear at my anus, my skin has a tear’. She said that and I knew that she had had anal sex so I took her and both of us came to the clinic and she got medication and she was well. …I told her, ‘You must tell the doctor directly that you are like this… they might check the wrong part, because normally they check the vagina’. … So they checked her and gave her medicine.

(*Female sex worker, Goroka*)

When we feel that our anus is itchy… we usually come into the clinic, and we say, ‘I want to check for STI and I came because I felt that my anus is itchy and also I have abdominal pains so I want to try and check whether I have an STI or not’. They would normally take it [blood] from our hands and check whether we have STIs or not, then they would give us gono-packs [packets of antibiotics for gonorrhoea], they used to give us.

(*Men who has sex with other men, Port Moresby*)

### Finding 6: There is support for self-collection of anal specimens

There was broad support for self-collection of anorectal specimen for STI testing, and a number of reasons were provided. Participants stated that they would be able to perform self-collection of specimen, and that the procedure would be easy.

We can do it and give them to check and give us results. It’s good. Like personally, I think this will be easier… I think we ourselves can do it at home that’s easy, we can do it at home.

(*Female sex worker, Goroka*)

[Self-collected] is alright. It’s alright, there is nothing wrong. It’s nice, It’s nice.

(*Transgender woman, Port Moresby*)

Self-collection was preferred as it was perceived to overcome concerns about sharing a private part of the body with a doctor. Research participants explained that there is not always both a male and female doctor at the clinic. Being able to collect the specimen themselves would overcome cultural inappropriateness of showing their bodies to a person of the other gender, particularly among female sex workers.

The anus is a body part where people get scared. I think it’s better we ourselves do the check and then take it to the doctor and give it to them and they will tell us the result.

(*Female sex worker, Goroka*)

The nurses and the doctors mainly take those tests, and they send them, so they see the results. So if you say we can do it ourselves then that’s alright too, its better… We can collect them [specimen] and give them to the doctor. That’s the same thing. Like if doctors are to check us, male doctors want check up. Women, that’s our custom in the highlands, we are scared of showing our bodies to them, so women can help us and it’s good that men can check men. Think about it in terms of that. If we ourselves can do the test and give it to the doctor, it’s much better.

(*Female sex worker, Goroka*)

### Finding 7: Some participants preferred to have a health worker collect specimens

Despite overwhelming support for the self-collection of specimen for anorectal STI testing, some participants explained that there should be an option for health workers to collect the sample. Reasons for this included illiteracy as a barrier to being able to understand instructions, fear of incorrect specimen collection, and a lack of privacy to collect the specimen at home.

For some who do not read and write it will be difficult, it will be difficult for them to see, they might make mistakes and tear their skin

(*Female sex worker, Goroka*)

Some will feel shy.

(*Transgender woman, Port Moresby*)

We might push that stick [swab] right in our anus. Things like that. So, it’s not good we do it ourselves. It’s good we can get a doctor will do this because that is what they learnt about and they know how to handle people and things like that.

(*Female sex worker, Goroka*)

The gender of the health worker is important. In addition to the quotes from female sex workers in the previous section, transgender women explained that they would prefer to seek assistance from a male health worker.

Because for the women, for us to come and access the service and talk to the women, we would feel like, because they are real girls, we feel ashamed.

(*Transgender woman, Port Moresby*)

Participants felt that having the flexibility to be able to collect a specimen at home, or with support from a health worker, wider participation in anorectal STI testing would be achieved.

We want, like if people want to check, like people like [health extension officer] can check us. Because we are similar to each other.

(*Transgender woman, Port Moresby*)

It is alright that it stays at the clinic and have the clinic people do it, and also there are people who can read and write and so they can take it and go and do it themselves. It depends on each person.

(*Female sex worker, Port Moresby*)

## Discussion

All participants were in favour of anal STI testing in PNG, and provided a range of reasons for their personal acceptance and willingness to undertake anal STI testing. There were no differences across the sample, either by population group or location. All participants were confident they could perform self-collection of specimens, although several stated that support from trained health workers should be available for other community members who may not feel comfortable with self-collection.

All participants supported the introduction of, and understood the personal benefits associated with, anorectal STI testing in PNG. While the majority of participants agreed that self-collection was preferable, there were minority concerns about literacy and confidence to collect the sample safely. That said, it was agreed that people should be given the option for self- or clinician-collection of anorectal specimen for STI testing.

While studies have been undertaken on the acceptability of self-collected swabs for vaginal STIs [20–23], few studies have explored self-collected swabs for anorectal STI testing [24–28], and little work with this focus has been undertaken in socio-culturally diverse low and middle-income country settings such as PNG. While acceptability of other public health interventions including medical male circumcision have been undertaken in PNG [29, 30], no acceptability studies have been undertaken on anorectal STI testing or the use of self-collected swabs for STI testing in PNG, or Pacific Asia more broadly. This is particularly important considering the stigma and shame associated with anal intercourse, the lack awareness of anorectal STIs, and the high burden of STIs amongst the populations involved in this study.

Study findings informed the inclusion of anorectal STI testing in a large bio-behavioural survey to be used to estimate anorectal STI prevalence among key populations in PNG for the first time [31]. This illustrates the importance and value gained from conducting qualitative research prior to biomedical and bio-behavioural research.

In addition to evidence that this study provides on acceptability of anorectal STI testing, this paper also highlights three other important issues. First, we have expanded the limited resource base documenting that anal sex is perceived to be common in PNG [7]. Second, we illustrate that given the correct research design and safe research settings, research participants can be encouraged to provide deep narratives about taboo issues that are often perceived as being too sensitive to discuss. Third, we have documented limited awareness about anorectal STIs among key populations in PNG. Without personal experience of anorectal symptoms, or sexual health promotion programs dealing with taboo and sensitive issues, people are vulnerable to misconceptions and misinformation about their sexual health. In PNG, anal intercourse is a topic that attracts much stigma and is largely ignored in sexual health programs. As a result, opportunities for people and communities to reduce the burden of STIs are lost.

The limitations of this study include the use of a small, unrepresentative sample of individuals from key populations in PNG, and that the research was conducted in only two settings. Despite these limitations, these findings have directly informed current bio-behavioural research with key populations that includes anorectal STI testing for the first time in PNG. As such, these findings contribute directly to research and policy on anal intercourse aimed at reducing the transmission of HIV and STIs, which is prioritised in PNG policies [32]. The study also highlighted high levels of engagement and interest from these populations in this taboo topic, and that there is a real willingness to explore future testing and create a demand for these testing services.

## Conclusions

In this exploratory study, findings suggest that a future anorectal STI testing program in PNG would be acceptable to female sex workers, men who have sex with men and transgender women, and would be important for enhancing people’s sexual health and preventing the negative health effects and ongoing transmission of anal STIs. Complementary research to establish the potential epidemiological impact, operational feasibility, cost-effectiveness and experiences of anorectal STI testing programs are required to inform future public health policy in this setting. Diagnosing and treating anorectal STIs (including asymptomatic cases), particularly among key populations should be an urgent priority in any future STI response program in PNG.

## Abbreviations

FGD: Focus group discussion
HIV: Human immunodeficiency virus
IMR: Institute of Medical Research
PNG: Papua New Guinea
STIs: Sexually transmitted infections
